# Concentrations of DDT metabolites in different food items and public health risk in Africa regions: systematic review and metal analysis

**DOI:** 10.3389/fpubh.2025.1511012

**Published:** 2025-04-02

**Authors:** Dechasa Adare Mengistu, Abraham Geremew, Roba Argaw Tessema, Tara Wolfing

**Affiliations:** ^1^School of Environmental Health, College of Health and Medical Science, Haramaya University, Harar, Ethiopia; ^2^School of Public Health, College of Health and Medical Science, Haramaya University, Harar, Ethiopia

**Keywords:** organochlorine pesticide, p,p'-DDT, p,p'-DDE, p,p'-DDD, health risk, Africa

## Abstract

**Background:**

DDT (Dichlorodiphenyltrichloroethane) is a synthetic organochlorine pesticide used in agriculture and mosquito control but later banned due to its harmful effects on humans. It persists in the environment, biomagnified through the food chain, and poses serious health risks, including reproductive defect, cancer, and nervous system disorders. DDT has a long half-life and potential of long-range transport and continuing to contaminate long after use and remains to pose a global environmental and health concern. Therefore, this review was designed to assess the concentration of DDT metabolites (p,p'-DDT, p,p'-DDD, and p,p'-DDE) in various food items and evaluate the health risk to consumers in the African.

**Methods:**

The preferred reporting item for systematic reviews and meta-analysis (PRISMA) protocol was used to conduct this work. SCOPUS, PubMed, Web of Science, Google Scholar, DOAJ, national repository, and MedNar were used to retrieve articles from October 1, 2023, to January 20, 2024. Meta-analysis data visualized using a forest plot. A random-effects model was applied when heterogeneity existed in overall mean concentration of DDT metabolites. The subgroup analysis, meta-regression, and sensitivity analysis was conducted and the Joanna Briggs Institute Critical Assessment tool to assess the quality of the studies.

**Results:**

The overall mean concentrations of p,p'-DDT, p,p'-DDD and p,p'-DDE, regardless of the types of food items, were 0.188, 0.22, and 0.0878 mg/kg, respectively. Based on the type of food items, The current study found that residue levels of DDT metabolites in vegetables, milk, and cereals exceeded the Maximum Residue Limits (MRLs) set by FAO/WHO/EU (0.05, 0.02, and 0.05 mg/kg, respectively), while residue levels in meat, khat, and fruit were below the MRLs (5, 0.5, and 0.05 mg/kg, respectively). The findings of the health risk assessment revealed that consumers are prone to both carcinogenic and non-carcinogenic risks from DDT metabolites. The persistence and bioaccumulation of these metabolites, along with multiple exposure routes and improper handling, may increase long-term health risks, even at low doses. DDT metabolite levels in most food categories exceed recommended limits, posing significant health risks to consumers. To reduce reliance on DDT, effective and cost-efficient alternative insecticides and vector-control strategies must be developed. Promoting environmental management, improving housing, and implementing farm-to-fork risk-based actions, such as Hazard Analysis and Critical Control Points (HACCP), across the food chain are crucial to mitigating the adverse effects of DDT exposure in the African region.

## 1 Introduction

The organochlorine insecticide DDT is one of the persistent organic pollutants (POPs) exempted from production restrictions under the Stockholm convention and humans' exposure continued through dietary exposure, drinking water contamination, indoor and outdoor personal airborne exposure, dermal exposure, and dust ingestion specifically among children are the main exposure pathways (1). The WHO has recommended its use in developing countries for indoor spraying to control malaria mosquitoes, primarily due to the lack of cost-effective alternatives with equivalent long-lasting residual efficacy and efficiency. The main challenge is balancing the high burden of malaria cases in endemic countries with the adverse environmental and health effects of DDT (2).

DDT and its metabolites are lipophilic (fat-soluble), accumulating in fat tissues and biomagnified through the food chain. They are absorbed through ingestion, inhalation, or dermal exposure, stored in fatty tissues like the liver and brain, and metabolized into DDE and DDD, which are toxic. Excretion occurs mainly through bile and feces, but elimination can take years due to their slow breakdown. Its residues can be detected in nearly every human body, posing long-term toxicity and a potential threat to human health by accumulating in all tissues, with the highest levels found in adipose tissue. It also poses risks to both terrestrial and marine ecosystems (3, 4). The FAO/WHO has reviewed DDT safety levels determining a specific NOAEL for humans is challenging because of inconsistent study findings. In animal studies, the NOAEL is about 0.05 mg/kg body weight per day, and the FAO/WHO/EU has set a provisional Acceptable Daily Intake (ADI) of 0.01 mg/kg body weight (5).

Evidence indicates that exposure to DDT is associated with adverse health outcomes, including breast cancer, diabetes, decreased semen quality, spontaneous abortion, endocrine disruption, acute neurological damage, and impaired neurodevelopment in children (4–11). The primary mechanism of DDT's action is that alter the proper function of the central nervous system as well as endocrine disruption by altering the molecular circuitry and function of the endocrine system (12). High exposure to DDT can still cause neurological symptoms like dizziness, headaches, nausea, and seizures.

DDT has a half-life of 2–15 years in soil and up to 150 years in aquatic sediments, and potential of long-range transport and continuing to contaminate long after use and continues to pose a global environmental and health concern, The use of DDT for unintended purposes and improper handling has resulted in high concentrations and bioaccumulation in environmental compartments (water, food, air, soil, and biota), endangering both human health and ecosystems (3). Comprehensive guidance and legal frameworks regarding the use, management, trade, and proper storage of DDT are provided by international organizations and conventions. However, these measures have not been strictly implemented to minimize the negative impact of DDT and its metabolites, or to reduce the burden of malaria, which is transmitted by indoor-resting vectors (5). The impact of DDT is worsening in developing countries, particularly in the African region, where inadequate knowledge and poor handling practices among users require urgent attention (13, 14). The unsafe and unintended application of DDT is a significant concern, posing serious threats in various parts of Africa (3, 14–16).

Several studies focus on the parent chemical DDT rather than its metabolites (9, 10), exploring the association between DDT exposure and specific health outcomes (17–21). However, there are scant systematic reviews and meta-analyses that have examined the pooled mean concentration of DDT metabolites (p,p'-DDT, p,p'-DDD, and p,p'-DDE) in various food items and the potential health risks they pose to consumers in the African region. The findings highlight a clear opportunity to design appropriate interventions to reduce the health risks posed to humans, animals, and the environment, particularly in Africa, where DDT use practices and awareness are inadequate.

## 2 Materials and methods

### 2.1 Eligibility criteria

The Population, Exposure, Comparator, and Outcome (PECO) criteria are used for the systematic review, and a comprehensive overview of the inclusion and exclusion criteria is provided below:-

#### 2.1.1 Types of population

We included quantitative studies on the concentration of DDT metabolites in various food items, such as vegetables, fruits, fish, meat, khat, milk, and cereals, without any restrictions on food types.

#### 2.1.2 Types of exposure

We included quantitative studies that evaluated the concentration of DDT metabolites in food items. These DDT metabolites were selected because they are primarily identified in the chosen food items, and the FAO/WHO and EU have set Maximum Residue Limits (MRL) for them.

#### 2.1.3 Type of comparator

The Maximum Residue Limits (MRLs) set by international standards (e.g., FAO/WHO and EU limits) or populations exposed to lower levels of DDT residues. This also considers the limits of quantification and detection, and to what extent food items contain DDT metabolites.

#### 2.1.4 Types of outcomes

Health risks, including both carcinogenic and non-carcinogenic risks, associated with exposure to concentration of DDT metabolites from food consumption.

#### 2.1.5 Types of studies

We included studies that assess the concentration of DDT metabolites in food items intended for human consumption. We excluded editorial papers, short communications, review articles, reports, preprints, qualitative studies, and articles with a high risk of bias. Articles published in English from 2010 to 2024 was included. The year 2010 was chosen as the starting point for the systematic review because those DDT metabolites of interest were extensively used in the African regions and we interested to investigate the recent concentrations of DDT metabolites in food items under reviewed.

### 2.2 Information sources and search strategy

The articles were retrieved from SCOPUS/Science Direct, PubMed/MEDLINE, Web of Science, Google Scholar, DOAJ, national repository and MedNar, from October 1, 2023, to January 20, 2024. A combination of Boolean logic operators (AND, OR, and NOT), Medical Subject Headings (MeSH) and main keywords were used to retrieve the articles from the included electronic databases. References within eligible studies were further screened for additional articles.

### 2.3 Study selection process

The study selection process was performed using the PRISMA flow chart, indicating the number of articles included in the study and excluded from the study with the major reasons of exclusion. Duplicate articles were removed using the ENDNOTE software version X5 (Thomson Reuters, USA). The authors independently screened the articles according to their titles and abstracts to determine their eligibility for the current study. The authors further independently evaluated the full texts of the relevant articles. Disagreements with respect to the inclusion and exclusion of articles were resolved by consensus. Finally, those studies that met the inclusion criteria were included in the current study.

### 2.4 Data extraction process

All authors separately extracted all relevant data required for the current study from eligible articles. To extract the data, a predetermined Microsoft Excel format (developed by the authors) consisting of study characteristics, including survey and publication year, a region where the study was conducted, sample size, and primary outcomes, residue or concentration of DDT metabolites (*p,p'-*DDT, *p,p'-*DDE and *p,p'-*DDD) in different food items, in African regions. Disagreement made with respect to data extraction was resolved through discussion.

### 2.5 Quality assessment

The included articles were subjected to quality evaluation by the authors using Joanna Briggs Institute Critical Assessment Tools (JBI) for the prevalence studies (22). The JBI critical appraisal tools with nine evaluation criteria includes:—(1) appropriate sampling frame; (2) proper sampling technique; (3) adequate sample size; (4) description of the study subject and setting description; (5) sufficient data analysis; (6) use of valid methods for the identified conditions; (7) valid measurement for all participants; (8) use of appropriate statistical analysis; and (9) adequate response rate. The articles were then evaluated by the authors to determine their eligibility. JBI critical appraisal tools have nine evaluation criteria. Each parameter was assigned a value of 1 if “Yes” and 0 if “No.” Based on the total score, each article was classified as low risk of bias (85% and above), moderate (60–85% score), or high risk of bias (60% and blow score). Finally, articles with moderate and low risk of bias were included in the study. Disagreements made between the authors regarding quality assessment were resolved by discussion after repeating the same procedures.

### 2.6 Statistical procedures and data analysis

The pooled mean concentration of DDT metabolites (p,p'-DDT, p,p'-DDE, and p,p'-DDD) among various food items was determined using the statistical software Comprehensive Meta-Analysis version 3.0. The meta-analysis data were visualized using a forest plot and a random-effects model was used. The I-squared test (*I*^2^ statistics) was used to evaluate the heterogeneity between articles. The level of heterogeneity was classified as without heterogeneity (0.0–25%), low heterogeneity (25–50%), moderate heterogeneity (50–75%), and high heterogeneity (>75%) (23). The random-effects model was used when the *I*^2^ statistic value is greater 75%, and a fixed-effects model otherwise.

Subgroup analysis was performed based on food categories, countries, and publication year. Meta-regression was performed to examine potential source of heterogeneity for each DDT metabolites using random effects model. A sensitivity analysis was performed to determine differences in pooled effects by dropping studies with largest, and/or smallest outcomes that were found to influence the overall concentration of DDT metabolites. The publication bias was assessed using a funnel plot.

### 2.7 Human health risk assessment

Health risk assessment estimates the probability of adverse health effects in human populations exposed to chemicals in contaminated environmental media (water, soil, air, and food) and assesses the impact of harmful contaminants over time. This review focuses on dietary exposure to three DDT metabolites (p,p'-DDT, p,p'-DDD, and p,p'-DDE) through food consumption. Non-carcinogenic and carcinogenic risks were estimated using the health risk assessment model provided by the United States Environmental Protection Agency (24) to evaluate the human health risks of DDT metabolites among food consumers in African regions.

#### 2.7.1 Non-carcinogenic risk estimation

The hazard quotient (HQ) and hazard index (HI or THQ) were used to estimate the non-carcinogenic effects of DDT metabolites. The estimated daily intake (EDI) was calculated using the following formula:

EDI = (Cm × EF × ED × AFC)/(ABW × ATE), where Cm represents the DDT metabolite concentration (mg/kg of dry weight), EF is the exposure frequency (365 days/year), ED is the exposure duration for carcinogenic risk (70 years), AFC is the average food consumption rate the per capita food consumption rate in Ethiopia is 0.887 kg/person/day (25), ABW is the average body weight of a consumer (70 kg), and ATE is the average time for carcinogenic exposure risk (life expectancy = 70 years × 365 days/year).

The hazard quotient (HQ) was estimated using the formula HQ = EDI/RfD, where EDI is the estimated daily intake of consumers in mg/day/kg of body weight, and RfD represents the oral reference dose (mg/kg/day) for each DDT metabolite (26).

The hazard index (HI) was estimated by adding the hazard quotient of each metabolite: HI = THQ = HQ _p,p′-DDT_ + HQ _p,p′-DDT_ + HQ _p,p′-DDT_, where the total hazard quotient (THQ) or hazard index (HI) accounts for the cumulative non-carcinogenic risk. This approach estimates the additive effects of exposure to multiple DDT metabolites simultaneously through food ingestion. There is a possibility of non-carcinogenic risk to consumers when the HI is >1, while consumers are unlikely to experience non-carcinogenic risk when the HI is <1

#### 2.7.2 Carcinogenic risk estimation

The risk of cancer to the consumer population from ingesting food items in the study area was evaluated using the Incremental Lifetime Cancer Risk (ILCR) model. The ILCR is calculated using the following formula:

ILCR = EDI × CSF, where EDI represents the estimated daily intake of DDT metabolites, and CSF represents the cancer slope factor for DDT metabolites in consumed food. The cancer slope factor (CSF) is a plausible upper-bound estimate of the probability that an individual will develop cancer from lifetime exposure to a chemical, typically over 70 years (26), and is expressed in units of mg/kg/day. Acceptable limits for carcinogenic health risks are within the range of 10^−6^ to 10^−4^, while a cancer risk (CR) >10^−4^ indicates a potential lifetime carcinogenic risk for consumers of the food items in Africa region.

## 3 Results

### 3.1 Study selection

**Figure 1:**
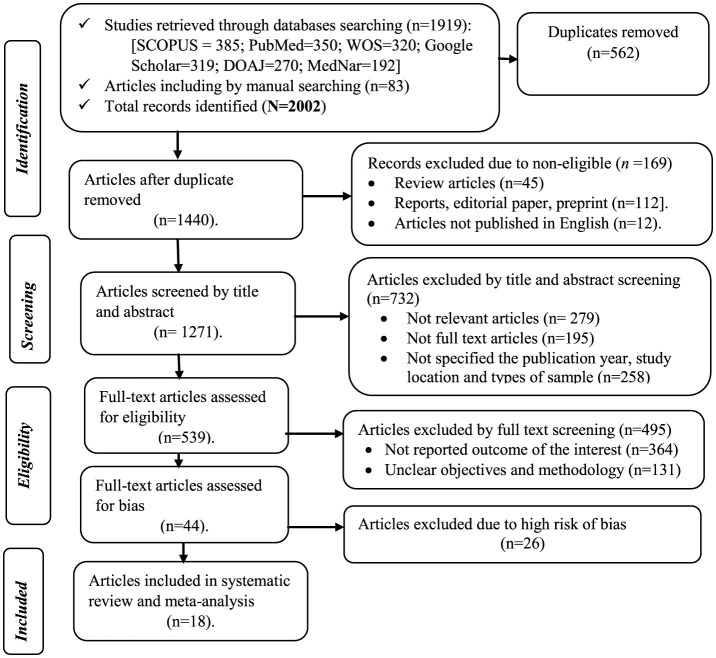
PRISMA flowchart of the study selection, 2024.

A total of 2002 studies were retrieved from the included databases and manual searches through Google. After retrieval, 562 duplicate articles were excluded. Furthermore, 169 studies were excluded due to being non-eligible, 732 were due to their title and abstract, 495 were due to full-text screening, and 26 articles were due to a high risk of bias. Finally, 18 articles were included in the current study (Figure 1).

### 3.2 General characteristics of the included articles

In the current study, 18 articles conducted in different African regions and met eligibility criteria were included. The included articles addressed the concentration of dichlorodiphenyltrichloroethane (DDT) metabolites (p,p'-DDT, p,p'-DDD, and p,p'-DDE) in 1 528 food and Khat samples between different types of food items and Khat consumed in the African region.

**Figure 2:**
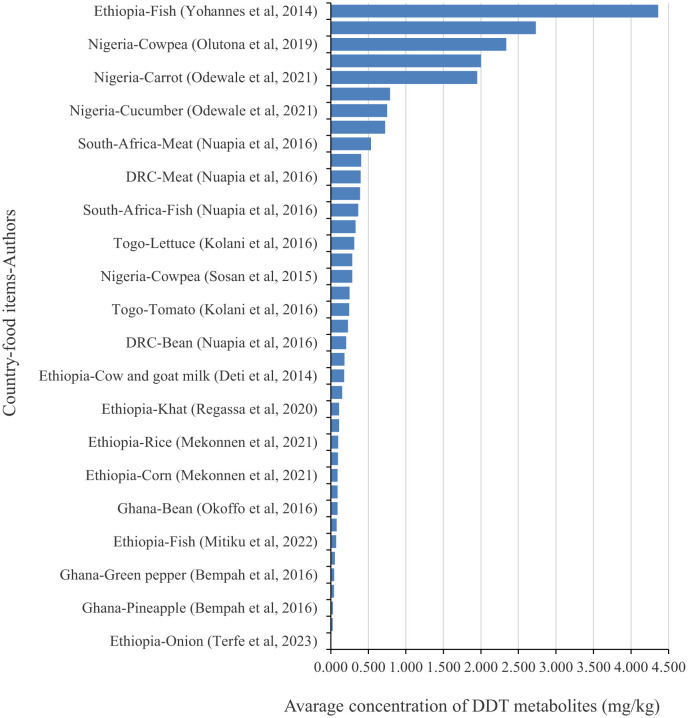
Average concentration of DDT metabolites by location, food items, and publication year.

Among the studies included in the current study, 8 (44.4%), 5 (27.8%), 1 (5.5%), 1 (5.5%), 1 (5.5%), 1 (5.5%), and 1 (5.5%) of the studies were conducted in Ethiopia (27–34), Nigeria (35–39), DRC (40), South Africa (40), Togo (41), Benin (42), and Ghana (43, 44), respectively. Among the studies that meet the eligibility criteria, 9 (50.0%), 5 (27.78%), 2 (11.1%), 2 (11.1%), 2 (11.1%), and 1 (5.5%) reported the concentration of DDT metabolites in vegetable (27, 30, 35, 36, 40–44), meat and fish (29, 33, 38–40), in fruit samples (35, 44), Milk (31, 32), Khat (34), and cereals (28), respectively (Supplementary Table 1). Relatively high average concentrations of DDT metabolites have been detected in Ethiopia, Nigeria, and South Africa (Figure 2).

### 3.3 Pesticide residues in different food items

#### 3.3.1 Concentration of p,p'-DDT residue in different food items

**Figure 3:**
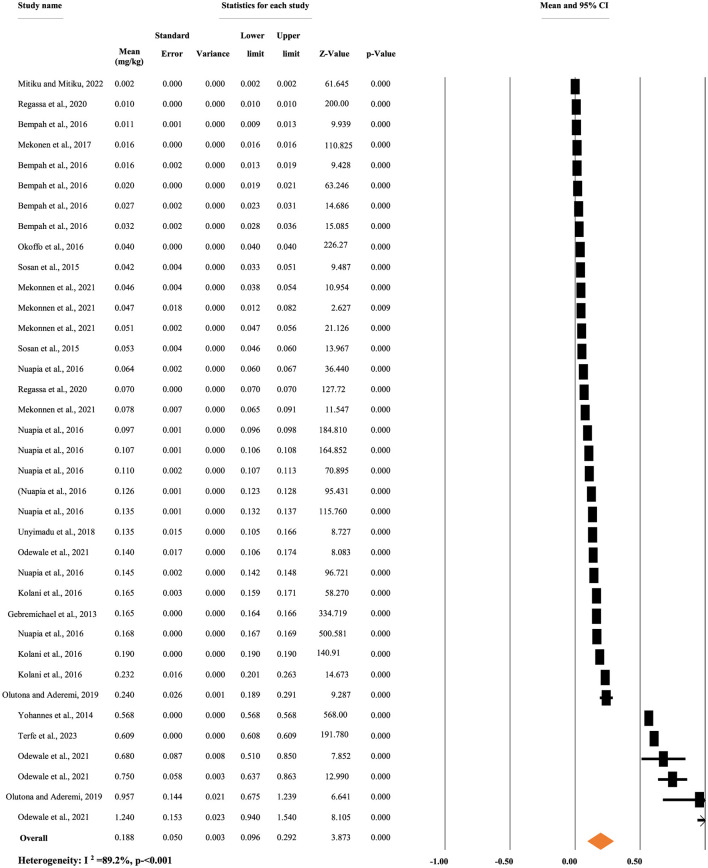
Overall mean concentration of p,p'-DDT residue regardless of the types of food items, in Africa.

Figure 2 shows the overall mean concentration of p,p'-DDT. A random-effects model was used to compute the overall mean concentration of each metabolite due to significant heterogeneity (*I*^2^ = 89.20%, *p* < 0.001). The overall mean concentration of p,p'-DDT, regardless of the types of food items, was 0.188 mg/kg, with a 95% CI of 9.6 to 29.2% and a *p* < 0.001 (Figure 3).

**Figure 4:**
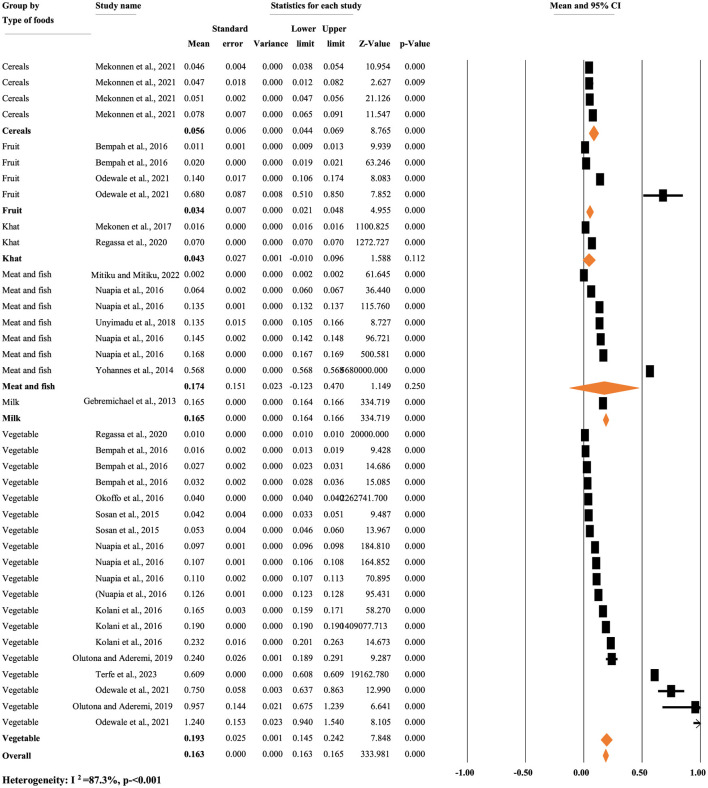
Mean concentration of p,p'-DDT residue based on types of food items, in Africa.

Figure 4 illustrates the mean concentration of p,p'-DDT residue based on types of food items. A random-effects model was used to calculate the overall mean concentration of each metabolite due to significant heterogeneity (*I*^2^ = 87.30%, *p* < 0.001). The subgroup analysis of p,p'-DDT based on the types of food items indicated that the mean concentration of p,p'-DDT in cereals, fruit, khat, meat and fish, milk, and vegetables accounted for 0.056, 0.034, 0.043, 0.174, 0.165, and 0.193 mg/kg, respectively (Figure 4).

#### 3.3.2 Concentration of p,p'-DDD residue in different food items

**Figure 5:**
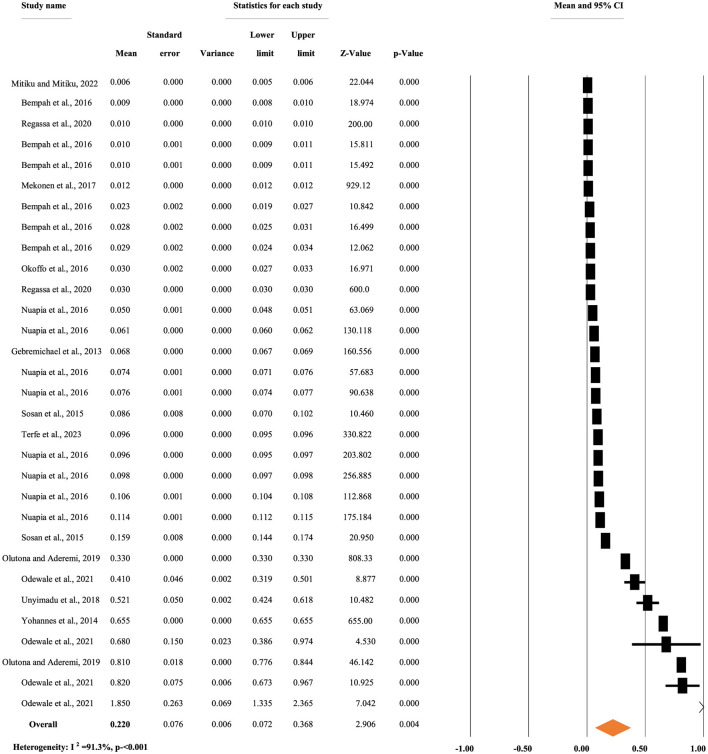
Overall mean concentration of p,p'-DDD residue regardless of the types of food items, in Africa.

Figure 5 depicts the overall mean concentration of p,p'-DDD residue, regardless of the types of food items. A random-effects model was used to compute the overall mean concentration of each metabolite due to significant heterogeneity (*I*^2^ = 91.30%, *p* < 0.001). The overall mean concentration of p,p'-DDD residue in different food items was 0.22 mg/kg, with a 95% CI of 7.2 to 36.8% and a *p* < 0.004 (Figure 5).

**Figure 6:**
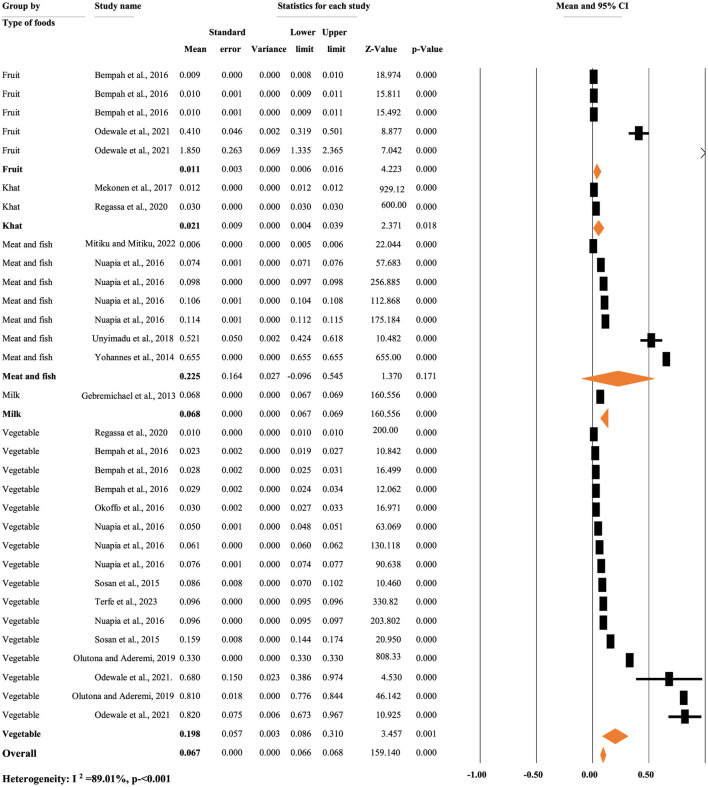
Pooled mean concentration of p,p'-DDD residue based on types of foods, in Africa.

Figure 6 illustrates the pooled mean concentration of p,p'-DDD residue based on types of food items. A random-effects model was used to compute the overall mean concentration of each metabolite due to significant heterogeneity (*I*^2^ = 89.01%, *p* < 0.001). The subgroup analysis of p,p'-DDD based on the types of food items indicated that the mean concentration of p,p'-DDD was 0.011, 0.021, 0.225, 0.068, and 0.198 mg/kg in fruit, khat, meat and fish, milk, and vegetables, respectively (Figure 6).

#### 3.3.3 Concentration of p,p'-DDE residue in different food items

**Figure 7:**
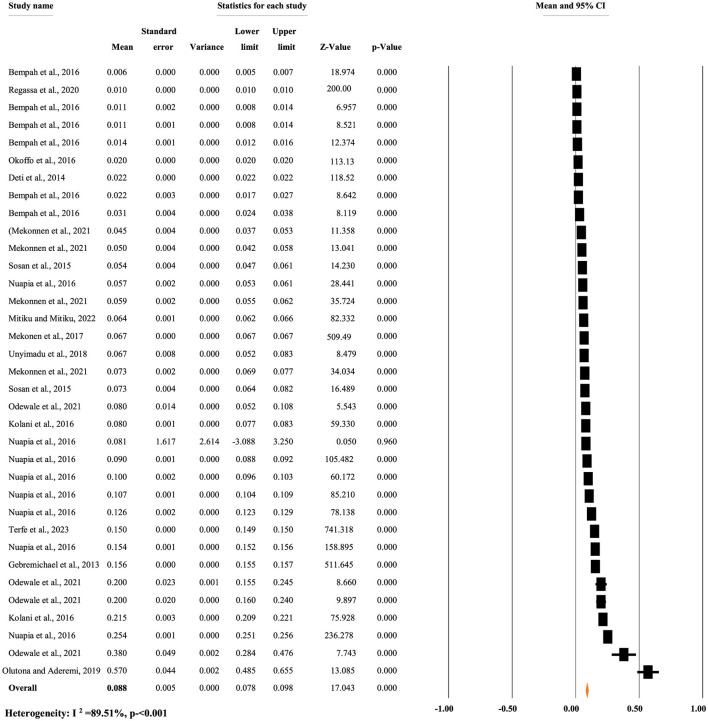
Overall mean concentration of p,p'-DDE residue regardless of the types of food items, in Africa.

Figure 7 reveals the overall mean concentration of p,p'-DDE residue, regardless of the types of food items. A random-effects model was used to compute the overall mean concentration of each metabolite due to significant heterogeneity (*I*^2^ = 89.51%, *p* < 0.001). The overall mean concentration of p,p'-DDE residue in different food items was 0.088 mg/kg, with a 95% CI of 7.8 to 9.8% and a *p* < 0.0001 (Figure 7).

**Figure 8:**
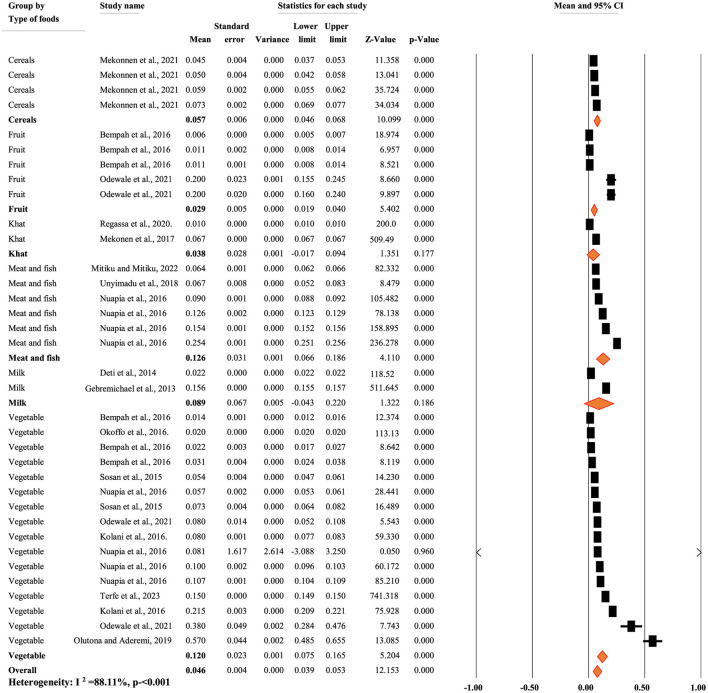
Mean concentration of pp DDE residue based on the types of food, in Africa.

Figure 8 illustrates the pooled mean concentration of p,p'-DDE residue based on types of food items. A random-effects model was used to compute the overall mean concentration of each metabolite due to significant heterogeneity (*I*^2^ = 88.11%, *p* < 0.001). The subgroup analysis of p,p'-DDE based on the types of food items revealed that the mean concentration of p,p'-DDE was 0.057, 0.029, 0.038, 0.126, 0.089, and 0.12 mg/kg in cereals, fruit, khat, meat and fish, milk, and vegetables, respectively (Figure 8).

### 3.4 Human health risk assessment

The current review findings indicates that consumers in the African region were exposed to higher health risks associated with the dietary intake of DDT metabolites through food consumption. The overall non-carcinogenic risk assessment from food items consumption was within unacceptable limits (HI = THQ > 1). Table 1 reveals that a THQ (HI) is ≥ 1 indicates a potential non-carcinogenic health risk and an ILCR value also >10^−4^ indicates potential lifetime carcinogenic risk. The ILCR value represents the likelihood of lifetime carcinogenic health risks from DDT metabolite through the consumption of food items (Table 1).

**Table 1:** Maximum concentrations (worst-case scenario) of DDT metabolites, along with non-carcinogenic and carcinogenic health risks associated with the dietary intake of DDT metabolites through food consumption in consumers in African regions.

**DDT metabolite**	**DDT metabolites concentration (mg/kg)**			**Non-carcinogenic risk estimation (mg/kg/day)**			**Carcinogenic risk estimation (mg/kg/day)**	
	**Mean** ±**SD**	**Min**	**Max**	**EDI**	**RfD**	**HQ**	**CSF**	**ILCR**
pp DDT	0.188 ± 0.047	0.141	0.235	2.98E-03	5.00E-04	5.96	3.40E-01	1.01E-03
pp DDD	0.22 ± 0.076	0.144	0.296	3.75E-03	3.00E-05	125.04	2.40E-01	9.00E-04
pp DDE	0.0878 ± 0.005	0.083	0.0929	1.18E-03	3.00E-04	3.92	3.40E-01	4.00E-04
					THQ = ∑HQ	134.92		

EF = 365 days; ED = 70 years; AFC = 0.887 kg/c/d average food consumption; ABW = 70 kg; ATE = 25,550 days (365 days × Life expectancy).

## 4 Discussion

The current study found that the residue of p,p'-DDT in milk (0.165 mg/kg) and cereals (0.056 mg/kg) exceeded the FAO/WHO MRL (0.02 mg/kg for milk) (45) and the EU MRL (0.04 mg/kg for milk; 0.05 mg/kg for cereals) (46). This indicates that consumers in African regions may be at high risk of exposure to p,p'-DDT through the consumption of contaminated food. In contrast, the residue of p,p'-DDT in meat (0.174 mg/kg) and khat (0.043 mg/kg) was lower than the FAO/WHO MRL (5 mg/kg for meat and 0.5 mg/kg for leaves and herbs) (45). This may be due to the limited number of studies providing data on these food categories.

The mean concentrations of p,p'-DDD in milk (0.0683 mg/kg) and vegetables (0.198 mg/kg) exceed the maximum recommended levels set by FAO/WHO (0.02 mg/kg for milk) (27) and the EU (0.04 mg/kg for milk; 0.05 mg/kg for vegetables) (46). These food categories are among the most commonly consumed in African regions, making consumers in Africa vulnerable to high levels of p,p'-DDD residues, which could pose significant health risks.

The current study found that the mean concentrations of p,p'-DDE residues in cereals (0.57 mg/kg), milk (0.089 mg/kg), and vegetables (0.1184 mg/kg) exceeded the FAO/WHO limits (0.1 mg/kg for cereals; 0.02 mg/kg for milk) (45) and the EU MRL limits (0.05 mg/kg for cereals, 0.04 mg/kg for milk, and 0.05 mg/kg for vegetables) (46). In contrast, the concentrations in fruit (0.029 mg/kg) and khat (0.0385 mg/kg) were below the EU MRL limits (0.05 mg/kg for fruit and 0.5 mg/kg for khat) (46).

The health risk assessment findings revealed that the hazard quotient of the three investigated DDT metabolites exceeds acceptable standards for both carcinogenic and non-carcinogenic health risk estimations. A similar finding were reported from a disease specific (diabetes) systematic review and meta-analysis of 43 studies conducted across America, Europe, Asia, and Africa examined the relationship between p,p'-DDT and p,p'-DDE concentrations and the risk of developing diabetes (47). The current review evidence indicates that higher concentrations of p,p'-DDT and p,p'-DDE were found in the reviewed food items, and the consumption of these food items with long-term exposure may pose an increased risk of diabetes and other chronic diseases. Limited knowledge about DDT metabolites, misuse of DDT, and poor handling practices increase non-carcinogenic health risks through short-term, high-level exposure, which many African consumers faced. Regional variations were observed in the concentration of total DDT metabolites, with the highest concentration detected in the eastern region of Africa (Ethiopia) in fish meat with 4.36 mg/kg, followed by the western region (Nigeria), with concentrations of 2.73 ± 0.98 mg/kg in watermelon and 2.337 ± 0.5027 mg/kg in cowpea. Because of the persistence and bioaccumulation tendency of these metabolites, combined with their potential synergistic effects and multiple exposure routes such as oral, dermal, and inhalation make consumers across all regions of Africa particularly vulnerable to lifetime carcinogenic risks from long-term, low-level exposure.

The current study revealed that the residue levels of DDT metabolites in vegetables, milk and cereals exceed the Maximum Residue Limits (MRLs) set by FAO/WHO and the EU. This indicates potential carcinogenic and non-carcinogenic health risks from exposure to these metabolites through food consumption. This underscores the need for effective law enforcement, food safety monitoring and evaluation, strengthened public health education on the health risks of DDT metabolites, and the application of farm-to-fork, risk-based actions, including hazard analysis and critical control points (HACCP), across the entire food chain to mitigate the adverse effects of DDT metabolite exposure in the African region.

Moreover, limiting the use of DDT under strict regulation to public health malaria control and the fight against insect-borne human diseases is a crucial medium-term intervention. The African regions should make efforts to reduce and eventually eliminate the use of DDT while minimizing the burden of malaria, as this is a fundamental long-term intervention. Alternative insecticides with equivalent efficacy, along with cost-effective and efficient vector-control strategies, must be developed to decrease reliance on DDT. Non-chemical approaches, such as environmental management and housing improvements (e.g., the use of window screens), should also be actively promoted as part of sustainable strategies for preventing vector-borne diseases, ultimately reducing the adverse effects of DDT and its metabolites on human health. DDT metabolites, such as DDE and DDD, accumulate in the body, disrupting hormonal balance (48, 49) and leading to reproductive disorders, neurotoxicity, and an increased risk of breast and liver cancers (50). Prenatal and early childhood exposure is linked to neurodevelopmental delays and impaired immune function (50). The endocrine-disrupting properties of DDT also contribute to infertility and metabolic disorders (51, 52), highlighting the need for stricter regulations and safer alternatives to reduce long-term exposure.

The body of research on DDT exposure highlights its detrimental effects on both neuro-degeneration and reproductive health. A study by Richardson et al. (53) found that elevated serum p,p'-DDE levels were linked to an increased risk of Alzheimer's disease (AD), suggesting that chronic exposure to DDT may contribute to cognitive decline. Additionally, a longitudinal study analyzing DDT levels in stored blood samples from 1959 to 1967 revealed that higher exposure during adolescence was associated with a 5fold increase in breast cancer risk later in life (54). This emphasizes DDT's role as an endocrine disruptor, contributing to hormone-related cancers. Furthermore, a comprehensive review conducted from 2003 to 2008 found that DDT exposure was linked to preterm birth and early weaning (11), while also providing evidence of its endocrine-disrupting effects on reproductive health.

DDT exposure also has a significant impact on fetal and male reproductive health. A study analyzing blood samples from pregnant women (1959–1966) found that higher maternal DDT levels were associated with preterm birth and lower birth weights (55), further supporting the harmful effects of DDT on fetal development. Additionally, a study examining sperm quality and hormone levels in men with high occupational and environmental DDT exposure (56) revealed a decline in sperm quality and potential infertility, confirming the negative effects of DDT on male fertility. Together, these findings highlight the widespread and harmful consequences of DDT exposure on human health.

DDT exerts its neurotoxic effects by targeting voltage-gated sodium channels in nerve cells, which are crucial for the transmission of electrical signals. When DDT binds to these channels, it prolongs their opening, leading to an excessive influx of sodium ions into the cells. This disruption prevents the channels from closing properly, resulting in uncontrolled neuronal firing and overstimulation, which underlies DDT's neurotoxicity. The activation and detoxification of DDT involve key enzymes such as Cytochrome P450 enzymes, Aryl hydrocarbon hydroxylase, and Acetylcholinesterase (AChE). The primary tissues affected by DDT include the nervous system, liver, fat tissue, and the reproductive, endocrine, and immune systems.

**Figure 9:**
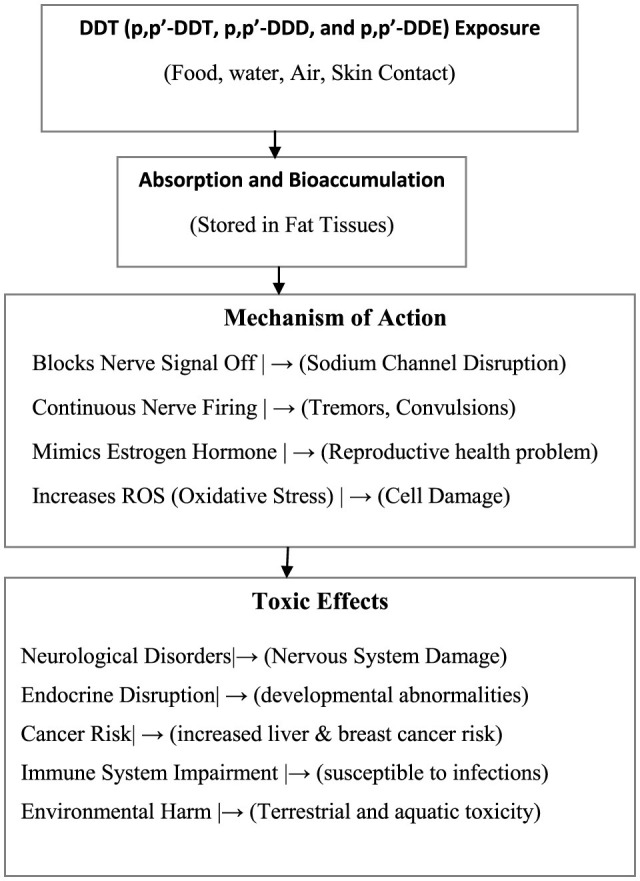
Diagram illustrate exposure and principal mechanism related to DDT toxicity.

The mechanism of DDT metabolite toxicity through food consumption begins with DDT accumulating in the environment, which then bioaccumulates in animals across the food chain. When humans consume DDT-contaminated food, the liver metabolizes DDT into metabolites like p,p'-DDD and p,p'-DDE. These metabolites can cause harmful effects, including endocrine disruption, neurological issues, and liver damage (Figure 9; Table 2). As a future direction, FAO/WHO and UNEP support the transition to safer pesticide alternatives, encouraging governments to phase out DDT in favor of integrated vector management (IVM) and adopt more sustainable methods for malaria control.

**Table 2:** Summary of DDT toxicity and mechanisms.

**DDT key aspect**	**Key characteristics**	**Principal mechanism**
Chemical nature	Lipophilic, Persists in the environment (long half-life)	Bioaccumulation in fatty tissues
Exposure routes	Ingestion, inhalation, dermal contact	Accumulates in body, mainly in fat
Neurotoxicity	Tremors, convulsions, dizziness	Interferes with Na+ channel closure
Endocrine effects	Reduced fertility, hormonal imbalance	Mimics estrogen, disrupts endocrine signaling
Carcinogenicity	Possible link to liver & breast cancer	Induces oxidative stress, DNA damage, and chronic effects
Ecological impact	Eggshell thinning in birds, aquatic toxicity	Biomagnification in food chains causes chronic exposure and effects

## 5 Strength and limitations

This study used multiple electronic databases with their applicable search strategies. Furthermore, the quality of the articles was evaluated using standard tools for quality assessment. Additionally, this work was done on the basis of the PRISMA guidelines or protocols. However, there was an unequal distribution of articles across African countries because a limited number of studies met eligible criteria. The DDT residue in different foods in many countries in the African Region was not included due to the limited number of studies that meet the eligibility criteria. Furthermore, due to a limited number of studies; systematic reviews and meta-analysis conducted on DDT residues in Africa and other regions, we are unable to adequately compare the findings of the current study with the findings of other studies. However, we have compared the pooled mean residue of DDT in different food types with a recommended maximum limit of FAO and WHO and the European Union to determine the health risk.

## 6 Conclusions

The concentration of DDT metabolites in fruits, meat and khat were not exceeded the maximum recommended levels set by FAO/WHO and the EU, while in vegetables, milk, and cereals exceeded, posing a potential health risk to consumers because of the synergistic effects of those metabolites and bioaccumulate tendency over the food chain. The health risk assessment revealed that consumers are prone to both carcinogenic and non-carcinogenic health risks from DDT metabolites.

Therefore, reducing reliance on DDT by using alternative insecticides with equivalent efficacy, along with cost-effective and efficient vector-control strategies is essential. Moreover, non-chemical approaches including environmental management and housing improvements, along with food safety interventions including public education and applying farm-to-fork risk-based actions, as well as monitoring and evaluation, to mitigate the adverse effects of DDT metabolite exposure from food consumption in the African region is recommended.

## Data Availability

The raw data supporting the conclusions of this article will be made available by the authors, without undue reservation.

## References

[1] Deziel NC Friesen MC Hoppin JA Hines CJ Thomas K Beane Freeman LE . A review of non-occupational pathways for pesticide exposure in women living in agricultural areas. Environ Health Perspect. (2015) 123:515–24. 10.1289/ehp.1408273 25636067 PMC4455586

[2] WHO. Demonstrating Cost Effectiveness and Sustainability of Environmentally Sound and Locally Appropriate Alternatives to DDT for Malaria Vector Control in Africa, 2009–2017. (2019). Geneva: The CODEX ALIMENTARIUS International Food Standard. Available online at: https://www.afro.who.int/sites/default/files/2019-03/AlternativeDDT-eng.pdf

[3] Kumari K Swamy S. Dichlorodiphenyltrichloroethane (DDT). Emerging Contaminants and Associated Treatment Technologies Pollutants of Global Concern. (2024). p. 31–48. 10.1007/978-3-031-50996-4_3

[4] Vall O Gomez-Culebras M Puig C Rodriguez-Carrasco E Baltazar AG Canchucaja L . Correction: prenatal and postnatal exposure to DDT by breast milk analysis in Canary Islands. PLoS ONE. (2018) 13:e0199904. 10.1371/journal.pone.0199904 29940037 PMC6016925

[5] FAO/WHO. CODEX ALIMENTARIUS International Food Standards. Pesticides Database. 2025 (2024). Rome. Available online at: https://openknowledge.fao.org/bitstreams/e96c7dbb-c396-43b3-a4c4-a1c2f84d7927/download (accessed December 10, 2024).

[6] WHO. Preventing Disease Through Healthy Environments: Exposure to Highly Hazardous Pesticides: A Major Public Health Concern. Geneva: World Health Organization (2019).

[7] Buah-Kwofie A Humphries MS Combrink X Myburgh JG. Accumulation of organochlorine pesticides in fat tissue of wild Nile crocodiles (*Crocodylus niloticus*) from iSimangaliso Wetland Park, South Africa. Chemosphere. (2018) 195:463–71. 10.1016/j.chemosphere.2017.12.084 29274992

[8] Gerber R Bouwman H Govender D Ishizuka M Ikenaka Y Yohannes YB . Levels of DDTs and other organochlorine pesticides in healthy wild Nile crocodiles (*Crocodylus niloticus*) from a flagship conservation area. Chemosphere. (2021) 264:128368. 10.1016/j.chemosphere.2020.128368 33007566

[9] Mew EJ Padmanathan P Konradsen F Eddleston M Chang S-S Phillips MR . The global burden of fatal self-poisoning with pesticides 2006–15: systematic review. J Affect Disord Sep. (2017) 219:93–104. 10.1016/j.jad.2017.05.002 28535450

[10] Thuy TT. Effects of DDT on environment and human health. J Educ Soc Sci. (2015) 2:108–4. Available online at: https://jesoc.com/wp-content/uploads/2015/11/SS-42.pdf

[11] Eskenazi B Chevrier J Rosas LG Anderson HA Bornman MS Bouwman H. The pine river statement: human health consequences of DDT use. Environ Health Perspect. (2009) 117:1359–67. 10.1289/ehp.11748 19750098 PMC2737010

[12] Coman G Farcas A Matei AV Florian C. Pesticides mechanisms of action in living organisms. In: Environmental Security Assessment and Management of Obsolete Pesticides in Southeast Europe. NATO Science for Peace and Security Series C: Environmental Security. Springer, Dordrecht (2013). p. 173–84. 10.1007/978-94-007-6461-3_16

[13] Afata TN Mekonen S Shekelifa M Tucho GT. Prevalence of pesticide use and occupational exposure among small-scale farmers in Western Ethiopia. Environ Health Insights. (2022) 16:11786302211072950. 10.1177/11786302211072950 35095275 PMC8793388

[14] Berni I Menouni A El IG Duca R-C Kestemont M-P Godderis L . Understanding farmers' safety behavior regarding pesticide use in Morocco. Sustain Prod Consum. (2021) 25:471–83. 10.1016/j.spc.2020.11.019

[15] Alebachew F Azage M Kassie GG Chanie M. Pesticide use safety practices and associated factors among farmers in Fogera district wetland areas, south Gondarzone, Northwest Ethiopia. PLoS ONE. (2023) 18:e0280185. 10.1371/journal.pone.0280185 36626384 PMC9831305

[16] Sheahan M Barrett CB Goldvale C. Human health and pesticide use in sub-Saharan Africa. Agric Econ. (2017) 48:27–41. 10.1111/agec.12384

[17] Stanganelli I De Felici MB Mandel VD Caini S Raimondi S Corso F . The association between pesticide use and cutaneous melanoma: a systematic review and meta-analysis. J Eur Acad Dermatol Venereol. (2020) 34:691–708. 10.1111/jdv.15964 31541557

[18] Chiu Y-H Sandoval-Insausti H Ley SH Bhupathiraju SN Hauser R Rimm EB . Association between intake of fruits and vegetables by pesticide residue status and coronary heart disease risk. Environ Int. (2019) 132:105113. 10.1016/j.envint.2019.105113 31473415 PMC6754761

[19] Karalexi MA Tagkas CF Markozannes G Tseretopoulou X Hernández AF Schüz J . Exposure to pesticides and childhood leukemia risk: a systematic review and meta-analysis. Environ Pollut. (2021) 285:117376. 10.1016/j.envpol.2021.117376 34380208

[20] Evangelou E Ntritsos G Chondrogiorgi M Kavvoura FK Hernández AF Ntzani EE . Exposure to pesticides and diabetes: a systematic review and meta-analysis. Environ Int. (2016) 91:60–8. 10.1016/j.envint.2016.02.013 26909814

[21] Kermani M Dowlati M Gholami M Sobhi HR Azari A Esrafili A . A global systematic review, meta-analysis and health risk assessment on the quantity of malathion, diazinon and chlorpyrifos in vegetables. Chemosphere. (2021) 270:129382. 10.1016/j.chemosphere.2020.129382 33418228

[22] The Joanna Briggs Institute (JBI). Critical appraisal tools for use in the JBI systematic reviews checklist for prevalence studies: The University of Adelaide. (2019). Available online at: https://joannabriggs.org/sites/default/files/2019-05/JBI_Critical_AppraisalChecklist_for_Prevalence_Studies2017_0.pdf (accessed December 10, 2024).

[23] Ades AE Lu G Higgins JP. The interpretation of random-effects meta-analysis in decision models. Med Decis Making. (2005) 25:646–54. 10.1177/0272989X05282643 16282215

[24] USEPA. Risk Assessment Guidance for Superfund Volume I Human Health Evaluation Manual (Part A) Interim Final. Office of Emergency and Remedial Response U.S. Environmental Protection Agency Washington, D.C (1989).

[25] Berhane G Paulos Z Tafere K Tamiru S. Foodgrain Consumption and Calorie Intake Patterns in Ethiopia. Development Strategy and Governance Division, International Food Policy Research Institute, Ethiopia Strategy Support Program II (ESSP II) ESSP II Working Paper No 23 (2011).

[26] ATSDR. Toxicological Profile for DDT, DDE, and DDD. Agency for Toxic Substances and Disease Registry and the U.S. Department of Health and Human Services. (2012). p. 369–70.

[27] Terfe A Mekonen S Jemal T. Pesticide residues and effect of household processing in commonly consumed vegetables in jimma zone, southwest Ethiopia. J Environ Public Health. (2023) 2023:7503426. 10.1155/2023/7503426 36755781 PMC9902158

[28] Mekonnen B Siraj J Negash S. Determination of pesticide residues in food premises using QuECHERS method in Bench-Sheko Zone, Southwest Ethiopia. Biomed Res Int. (2021) 2021:6612096. 10.1155/2021/6612096 33829061 PMC8004370

[29] Mitiku BA Mitiku MA. Organochlorine pesticides residue affinity in fish muscle and their public health risks in North West Ethiopia. Food Sci Nutr. (2022) 10:4331–8. 10.1002/fsn3.3025 36514750 PMC9731529

[30] Regassa C Tolcha T Gomoro K Megersa N. Determination of residue levels of DDT and its metabolites in khat and cabbage samples using QuEChERS sample preparation method combined with GC-MS detection. Ethiop J Sci Sustain Dev. (2020) 7:44–53. 10.20372/ejssdastu:v7.i1.2020.119

[31] Gebremichael S Birhanu T Tessema DA. Analysis of organochlorine pesticide residues in human and cow's milk in the towns of Asendabo, Serbo and Jimma in South-Western Ethiopia. Chemosphere. (2013) 90:1652–7. 10.1016/j.chemosphere.2012.09.008 23062941

[32] Deti H Hymete A Bekhit AA Mohamed AMI Bekhit AE-DA. Persistent organochlorine pesticides residues in cow and goat milks collected from different regions of Ethiopia. Chemosphere. (2014) 106:70–4. 10.1016/j.chemosphere.2014.02.012 24630448

[33] Yohannes YB Ikenaka Y Nakayama SM Ishizuka M. Organochlorine pesticides in bird species and their prey (fish) from the Ethiopian Rift Valley region, Ethiopia. Environ Pollut. (2014) 192:121–8. 10.1016/j.envpol.2014.05.007 24907858

[34] Mekonen S Ambelu A Negassa B Spanoghe P. Exposure to DDT and its metabolites from khat (*Catha edulis*) chewing: consumers risk assessment from southwestern Ethiopia. Regul Toxicol Pharmacol. (2017) 87:64–70. 10.1016/j.yrtph.2017.05.008 28483709

[35] Odewale GO Sosan MB Oyekunle JAO Adeleye AO. Human health risk assessment of dichlorodiphenyltrichloroethane (DDT) and hexachlorocyclohexane (HCH) pesticide residues in fruits and vegetables in Nigeria. Environ Sci Pollut Res. (2021) 28:33133–45. 10.1007/s11356-021-12747-7 33638082

[36] Olutona G Aderemi M. Organochlorine pesticide residue and heavy metals in leguminous food crops from selected markets in Ibadan, Nigeria. Legume Sci. (2019) 1:1–9. 10.1002/leg3.3

[37] Sosan M Oyekunle J Olufade Y. Dichloro-diphenyl-trichloro-ethane (DDT) and hexachlorohexane (HCH) pesticide residues in foodstuffs from markets in Ile-Ife, Nigeria. Int J Biol Chem Sci. (2015) 9:442–53. 10.4314/ijbcs.v9i1.38

[38] Tongo I Ezemonye L. Human health risks associated with residual pesticide levels in edible tissues of slaughtered cattle in Benin City, Southern Nigeria. Toxicol Rep. (2015) 2:1117–35. 10.1016/j.toxrep.2015.07.008 28962453 PMC5598159

[39] Unyimadu JP Osibanjo O Babayemi JO. Levels of organochlorine pesticides in brackish water fish from Niger River, Nigeria. J Environ Public Health. (2018) 2018:2658306. 10.1155/2018/2658306 30050580 PMC6046123

[40] Nuapia Y Chimuka L Cukrowska E. Assessment of organochlorine pesticide residues in raw food samples from open markets in two African cities. Chemosphere. (2016) 164:480–7. 10.1016/j.chemosphere.2016.08.055 27614040

[41] Kolani L Mawussi G Sanda K. Assessment of organochlorine pesticide residues in vegetable samples from some agricultural areas in Togo. American Journal of Analytical Chemistry. (2016) 7:332–41. 10.4236/ajac.2016.74031

[42] Agnandji P Ayi-Fanou L Gbaguidi MA Cachon BF Hounha M Dikpo MT . Distribution of organochlorine pesticides residues in *Solanum macrocarpum* and *Lactuca sativa* cultivated in South of Benin (Cotonou and Seme-Kpodji). Am J Anal Chem. (2018) 6:19–25. 10.12691/ajfst-6-1-4

[43] Okoffo ED Fosu-Mensah BY Gordon C. Persistent organochlorine pesticide residues in cocoa beans from Ghana, a concern for public health. Int J Food Contam. (2016) 3:1–11. 10.1186/s40550-016-0028-4

[44] Bempah CK Agyekum AA Akuamoa F Frimpong S Buah-Kwofie A. Dietary exposure to chlorinated pesticide residues in fruits and vegetables from Ghanaian markets. J Food Compos Anal. (2016) 46:103–13. 10.1016/j.jfca.2015.12.001

[45] FAO/WHO. The Codex Alimentarius International Food Standards. Available onlibe at: https://www.fao.org/fao-who-codexalimentarius/codex-texts/dbs/pestres/pesticide-detail/ru/?p_id=21 (accessed February 2024).

[46] EU. Commission Regulation (EU) 2023/163 of 18 January 2023 amending Annexes II and III to Regulation (EC) No 396/2005 of the European Parliament and of the Council as regards maximum residue levels for DDT and oxathiapiprolin in or on certain products (Text with EEA relevance). (2023). Available onlibe at: https://eur-lex.europa.eu/eli/reg/2023/163/oj (accessed December 8, 2024).

[47] Yipei Y Zhilin L Yuhong L Meng W Huijun W Chang S . Assessing the risk of diabetes in participants with DDT DDE exposure- a systematic review and meta-analysis. Environ Res. (2022) 210:113018. 10.1016/j.envres.2022.113018 35227676

[48] Frye C Bo E Calamandrei G Calza L Dessì-Fulgheri F Fernández M . Endocrine disrupters: a review of some sources, effects, and mechanisms of actions on behaviour and neuroendocrine systems. J Neuroendocrinol. (2012) 24:144–59. 10.1111/j.1365-2826.2011.02229.x 21951193 PMC3245362

[49] Bergman Å Heindel JJ Jobling S Kidd K Zoeller TR Organization WH. State of the Science of Endocrine Disrupting Chemicals 2012. Geneva: World Health Organization (2013). 10.1016/j.toxlet.2012.03.020

[50] Tadevosyan NS Kirakosyan GV Muradyan SA Poghosyan SB Khachatryan BG. Relationship between respiratory morbidity and environmental exposure to organochlorine pesticides in armenia. Journal of Health Pollution. (2021) 11:210904. 10.5696/2156-9614-11.31.210904 34434596 PMC8383794

[51] Freire C Koifman RJ Sarcinelli PN Rosa ACS Clapauch R Koifman S. Association between serum levels of organochlorine pesticides and sex hormones in adults living in a heavily contaminated area in Brazil. Int J Hyg Environ Health. (2014) 217:370–8. 10.1016/j.ijheh.2013.07.012 23972672

[52] Wahlang B. Exposure to persistent organic pollutants: impact on women's health. Rev Environ Health. (2018) 33:331–48. 10.1515/reveh-2018-0018 30110273

[53] Richardson JR Roy A Shalat SL von Stein RT Hossain MM Hossain MM . Elevated serum pesticide levels and risk for Alzheimer disease. JAMA Neurol. (2014) 71:284–90. 10.1001/jamaneurol.2013.6030 24473795 PMC4132934

[54] Cohn BA Cirillo PM Sholtz RI Wolff MS. DDT and breast cancer: Cohn et al. respond. Environ Health Perspect. (2008) 116:A153–4. 10.1289/ehp.11025R 18414606 PMC2290990

[55] Longnecker MP Klebanoff MA Zhou H Brock JW. Association between maternal serum concentration of the DDT metabolite DDE and preterm and small-for-gestational-age babies at birth. Lancet. (2001) 358:110–4. 10.1016/S0140-6736(01)05329-6 11463412

[56] Ayotte P Giroux S Dewailly É Hernánde A Mauricio F Paulina D . DDT spraying for malaria control and reproductive function in mexican men. Epidemiology. (2001) 12:366–7. 10.1097/00001648-200105000-00022 11338320

