# Supplementary material for: Concentrations of DDT metabolites in different food items and public health risk in Africa regions: systematic review and metal analysis
Source: Front Public Health. 2025 Apr 2;13:1511012. doi: 10.3389/fpubh.2025.1511012 (PMC12000096; doi:10.3389/fpubh.2025.1511012)
Supplement: Supplementary file 1 [file Table_1.doc]

***Supplementary Table 1***: General characteristics of the studies included in the study, 2024

| Food category | Types of food items | Sample size | Publication year | Types of pesticides and its concentration in mg/kg | | | | | Country | | Reference |
| --- | --- | --- | --- | --- | --- | --- | --- | --- | --- | --- | --- |
|  |  |  |  | Pp DDT ± SD | Pp DDD± SD | Pp DDE± SD | Total DDT |  | |  | |
| Vegetable | Bean | 20 | 2016 | 0.0967± 0.0023 | 0.0495± 0.0035 | 0.0566 ± 0.0089 | 0.2028±0.0147 | DRC | | (40) | |
|  | Bean | 20 | 2016 | 0.1097± 0.0069 | 0.0758 ±0.0037 | 0.0997± 0.0074 | 0.2852±0.0180 | South Africa | | (40) | |
|  | Bean | 32 | 2016 | 0.04±0 | 0.03±0.01 | 0.02±0 | 0.09±0.01 | Ghana | | (43) | |
|  | Cabbage | 4 | 2020 | 0.01 ±0 | 0.01±0 | 0.004±0 | 0.024±0 | Ethiopia | | (30) | |
|  | Cabbage | 20 | 2016 | 0.1069 ± 0.0029 | 0.0611 ±0.0021 | 0.0811 ± 7.23 | 0.2491±0.2350 | DRC | | (40) | |
|  | Cabbage | 20 | 2016 | 0.1259 ± 0.0059 | 0.0957± 0.0021 | 0.1067 ± 0.0056 | 0.3283±0.0136 | South Africa | | (40) | |
|  | Cabbage | 50 | 2016 | 0.016 ± 0.012 | 0.023 ± 0.015 | 0.014 ± 0.008 | 0.053±0.035 | Ghana | | (44) | |
|  | Cabbage | 55 | 2016 | 0.190 ± 0.000 | -- | 0.215 ± 0.021 | 0.405±0.021 | Togo | | (41) | |
|  | Tomato | 50 | 2016 | 0.027 ± 0.013 | 0.029 ± 0.017 | 0.022 ± 0.018 | 0.078±0.048 | Ghana | | (44) | |
|  | Tomato | 12 | 2021 | 1.24±0.53 | 0.68±0.52 | 0.08±0.05 | 2±0.58 | Nigeria | | (35) | |
|  | Tomato | 55 | 2016 | 0.165 ± 0.021 | -- | 0.080 ± 0.010 | 0.245±0.031 | Togo | | (41) | |
|  | Carrot | 12 | 2021 | 0.75±0.20 | 0.82±0.26 | 0.38±0.17 | 1.95±0.63 | Nigeria | | (35) | |
|  | Lettuce | 50 | 2016 | 0.032 ±0.015 | 0.028 ±0.012 | 0.031± 0.027 | 0.091±0.054 | Ghana | | (44) | |
|  | Lettuce | 40 | 2016 | 0.232 ± 0.1 | -- | 0.081 ± 0.047 | 0.313±0.147 | Togo | | (41) | |
|  | Cowpea | 6 | 2019 | 0.957±0.353 | 0.81±0.043 | 0.57±0.1067 | 2.337±0.5027 | Nigeria | | (36) | |
|  | Bean | 6 | 2019 | 0.24±0.0633 | 0.33±0 | 0.22±0.0.047 | 0.79±0.0633 | Nigeria | | (36) | |
|  | Yam chips | 10 | 2015 | 0.042 ± 0.014 | 0.086 ± 0.026 | 0.054 ± 0.012 | 0.182±0.04 | Nigeria | | (37) | |
|  | Onion | 12 | 2023 | 0.6085± 0.0001 | 0.0955± 0.001 | 0.1498± 0.0007 | 0.00213 | Ethiopia | | (27) | |
|  | Cowpea | 10 | 2015 | 0.053+0.012 | 0.159+ 0.024 | 0.073+0.014 | 0.285±0.05 | Nigeria | | (37) | |
| Fruit | Mango | 40 | 2016 | 0.020 ± 0.002 | 0.010 ± 0.004 | 0.011± 0.010 | 0.041±0.016 | Ghana | | (44) | |
|  | Cucumber | 12 | 2021 | 0.14±0.06 | 0.41±0.16 | 0.20±0.08 | 0.75±0.3 | Nigeria | | (35) | |
|  | Pineapple | 40 | 2016 | 0.011 ± 0.007 | 0.009 _ 0.003 | 0.006 ±0.002 | 0.026±0.012 | Ghana | | (44) | |
|  | Green pepper | 60 | 2016 | 0.024 ± 0.012 | 0.010±0.005 | 0.011 ± 0.010 | 0.045±0.027 | Ghana | | (44) | |
|  | Watermelon | 12 | 2021 | 0.68±0.30 | 1.85±0.91 | 0.20±0.07 | 2.73±0.98 | Nigeria | | (35) | |
| Milk | Cow milk | 30 | 2013 | 0.165±0.0027 | 0.0683±0.0023 | 0.156±0.0017 | 0.3893±0.0067 | Ethiopia | | (31) | |
|  | Cow and goat milk | 30 | 2014 | 0.155±0 | -- | 0.02164±0 | 0.17664±0 | Ethiopia | | (32) | |
| Cereals | Corn | 30 | 2021 | 0.046 ± 0.023 | -- | 0.045 ± 0.0217 | 0.091±0.0447 | Ethiopia | | (28) | |
|  | Rice | 30 | 2021 | 0.047 ± 0.098 | -- | 0.05 ± 0.021 | 0.097±0.021 | Ethiopia | | (28) | |
|  | Sorghum | 30 | 2021 | 0.0513 ± 0.0133 | -- | 0.0587 ± 0.009 | 0.11±0.0223 | Ethiopia | | (28) | |
|  | Common millet | 30 | 2021 | 0.078 ± 0.037 | -- | 0.0727 ± 0.0117 | 0.1507±0.0487 | Ethiopia | | (28) | |
| Meat and fish | Meat | 20 | 2016 | 0.1451± 0.0067 | 0.0976 ± 0.0017 | 0.1542 ± 0.0043 | 0.3969±0.0084 | DRC | | (40) | |
|  | Fish | 20 | 2016 | 0.0637 ± 0.0078 | 0.0735 ± 0.0057 | 0.0901 ± 0.0038 | 0.2273±0.0173 | DRC | | (40) | |
|  | Fish | 137 | 2022 | 0.0016 ± 0.0003 | 0.0056 ±0.003 | 0.0640 ± 0.0091 | 0.0712±0.0124 | Ethiopia | | (29) | |
|  | Fish | 100 | 2014 | 0.568±0 | 0.655±0 | 3.138±0 | 4.361±0 | Ethiopia | | (33) | |
|  | Fish | 60 | 2018 | 0.1352 ± 0.12 | 0.521 ± 0.385 | 0.0671 ± 0.0613 | 0.7233±0.5663 | Nigeria | | (39) | |
|  | Meat | 20 | 2016 | 0.1679 ± 0.0015 | 0.1136 ± 0.0029 | 0.2536 ± 0.0048 | 0.5351±0.0092 | South Africa | | (40) | |
|  | Fish | 20 | 2016 | 0.1346 ± 0.0052 | 0.106 ± 0.0042 | 0.1258 ± 0.0072 | 0.3664±0.0166 | South Africa | | (40) | |
| Khat | Khat | 4 | 2020 | 0.07±0 | 0.03 ±0 | 0.01±0 | 0.11±0 | Ethiopia | | (30) | |
|  | Khat | 58 | 2017 | 0.0159±0 | 0.0122±0 | 0.0669±0 | 0.095±0 | Ethiopia | | (34) | |
